# Immunosenescence, inflammation and Alzheimer’s disease

**DOI:** 10.1186/2046-2395-1-8

**Published:** 2012-11-01

**Authors:** Adriana Martorana, Matteo Bulati, Silvio Buffa, Mariavaleria Pellicanò, Calogero Caruso, Giuseppina Candore, Giuseppina Colonna-Romano

**Affiliations:** 1 Immunosenescence Unit, Department of Pathobiology and Medical and Forensic Biotechnologies, University of Palermo, corso Tukory 211, 90134, Palermo, Italy; 2 Department of Internal Medicine II, Center for Medical Research, Tübingen Aging and Tumor Immunology Group, University of Tübingen, Waldhörnlestraße 22, 72072, Tübingen, Germany

**Keywords:** Immunosenescence, Alzheimer’s disease, Inflammation, Cytokine, Chemokine, Lymphocyte, Ageing

## Abstract

Ageing impacts negatively on the development of the immune system and its ability to fight pathogens. Progressive changes in the T-cell and B-cell systems over the lifespan of individuals have a major impact on the capacity to respond to immune challenges. The cumulative age-associated changes in immune competence are termed immunosenescence that is characterized by changes where adaptive immunity deteriorates, while innate immunity is largely conserved or even upregulated with age. On the other hand, ageing is also characterized by “inflamm-ageing”, a term coined to explain the inflammation commonly present in many age-associated diseases. It is believed that immune inflammatory processes are relevant in Alzheimer’s disease, the most common cause of dementia in older people. In the present paper we review data focusing on changes of some immunoinflammatory parameters observed in patients affected by Alzheimer’s disease.

## Review

### Ageing and the immune system

During the past century, humans have gained more years of average life expectancy than in the last 10,000 years. Currently, people are living much longer than they used to; and the longer they live, the longer their bodies are exposed to environmental factors that increase the risk of age-associated diseases. The reduction of the response to environmental stimuli is associated with an increased inclination towards illness and death. In western countries, the mortality rate increases in people over 65 years old, if compared with younger individuals, by 100-fold for stroke or chronic lung disease, by 92-fold for heart disease, by 89-fold for influenza and correlated pneumonia infections, and by 43-fold for cancer [1]. Ageing is the consequence of the collapse of self-organizing systems and reduced ability to adapt to the environment, and it has been suggested that normal human ageing is associated with a loss of complexity in a variety of anatomic structures and physiological processes [2]. These losses lead to physical inability, impaired mental functional capacity and organ and apparatus deregulation [3], with the consequence of increased susceptibility to diseases and death. On the contrary, healthy ageing seems directly correlated with a good functioning of the immune system, suggesting that it is related to both environmental factors and genetic background. Indeed, many studies have focused on genetic determinants of longevity in genes regulating the immune-inflammatory response [4-7].

Ageing impacts negatively on the development of the immune system and its ability to function. Progressive changes in the T-cell and B-cell systems over the lifespan of individuals have a major impact on the capacity to respond to immune challenges. These cumulative age-associated changes in immune competence are termed immunosenescence. According to the remodeling theory of ageing proposed several years ago [8], the current data on human immunosenescence describe a complex scenario where adaptive immunity deteriorates, while innate immunity is largely conserved or even up-regulated with age. Under an evolutionary perspective, antigens are the cause of a persistent lifelong antigenic stress, responsible for the accumulation of effector CD8^+^/CD28^-^ T cells, the decrease of naïve T lymphocytes (CD45RA^+^CD62L^+^) and the marked shrinkage of the T-cell repertoire with age [9-14]. The humoral compartment is also affected in the aged [15-20]; indeed, B-cell numbers are decreased and the B-cell repertoire is influenced by ageing through the quality of antibody response [21-25], and this decreased B-cell diversity is associated with poor health status [26-28]. Immunosenescence is thus not a random deteriorative phenomenon, as was hypothesized in 1989 in “the network theory of aging”, but could be envisaged as the result of the continuous challenge of the unavoidable exposure to a variety of potential antigens such as viruses and bacteria, but also food and self-molecules among others [12,13,29-31].

Immunosenescence therefore materially contributes to the decreased ability of the older person to control infectious diseases, which is also reflected in the observed poor response to vaccination [25,32-34]. In recent years, the idea of the immunological risk phenotype (IRP) that includes some immunological parameter changes that predict survival has been suggested [35-37]. A good immune system in the older person is tightly correlated to health status, and, as aforementioned, some immunological parameters are often markedly reduced in these subjects (Table 1). On the contrary, infectious diseases, cancer, autoimmune diseases and inflammatory chronic diseases such as atherosclerosis, heart diseases and Alzheimer’s disease (AD) are frequent in this phase of life [38]. Indeed, much experimental and clinical evidence has suggested that the immune system is implicated, with a variable degree of importance, in almost all age-related or associated diseases.

**Table 1:** Modifications of T-cell and B-cell systems in older humans

**T cells and B cells or B-cell products**	**Lymphocyte subpopulations**	**Change**	**Reference**
CD3^+^, CD3^+^CD4^+^, CD3^+^CD8^+^	Total T cells, T helper cells,	Decrease	[9]
(percentage and absolute number)	cytotoxic T lymphocytes		[14]
CD3^+^CD45RA^+^CD62L^+^	Naïve T cells	Decrease	[10]
(percentage)			[11]
			[12]
			[13]
CD8^+^CD28^-^	Effector T cells	Increase	[10]
(percentage)			[11]
			[12]
			[13]
CD19^+^	Total B cells	Decrease	[24]
(percentage and absolute number)			[25]
			[16]
			[17]
			[18]
CD19^+^CD5^+^	B1 cells	Decrease	[15]
(percentage and absolute number)			
CD19^+^IgD^+^CD27^–^	Naïve B cells	Decrease	[19]
(percentage)			
CD19^+^IgD^-^CD27^–^	Double Negative B cells	Increase	[19]
(percentage)			[24]
			[20]
IgG, IgA		Increase	[21]
		No change	[22]
IgD, IgM		Decrease	[21]
IgE		No change	[21]
(after specific immunization)			[22]
		Decrease	[23]
Autoantibodies		Increase	[27]
			[26]

Ageing is accompanied by a chronic low-grade inflammatory state demonstrated by the increased serum levels of inflammatory mediators such as cytokines and acute phase proteins in the aged [39,40]. The most important role in this basal pro-inflammatory status in the older person seems to be played by chronic antigenic stress, which, interacting with the genetic background, potentially triggers the onset of age-related inflammatory diseases [6,7,41]. The inflammatory process is a physiological phenomenon that is necessary for the elimination of pathogenic viruses or bacteria, but the prolonged period to which aged people are exposed may lead to chronic inflammation that inevitably damages several organs. Chronic inflammation appears to be involved in the pathogenesis of all age-related diseases such as AD, atherosclerosis, diabetes, sarcopenia and cancer [4,42-47].

### Inflammation, Alzheimer’s disease and immune response

AD is the most common cause of dementia in older people and it is estimated that 27 million people are affected worldwide [48,49]. As the life expectancy of the population increases, the number of affected individuals is predicted to triple by 2050 [49,50]. Age is therefore the main risk factor in AD, although early-onset disease can occur before age 60. AD may not be an inevitable occurrence of the aging process, but it is a disease with significant genetic roots. Indeed, genetics is important not only in predicting susceptibility but also the age of disease onset in the older person [51]. Other important risk factors are environmental events in early life as well as childhood IQ [52] and gender. In most studies, women were found to be at greater risk for AD. However, it is not clear whether this effect is due to genetic or hormonal differences between males and females or whether it is a surrogate marker of other still unmeasured socioeconomic factors [53].

AD is a progressive brain disorder affecting regions of the brain that control memory and cognitive functions. The two major neuropathologic hallmarks of AD are extracellular amyloid-beta (Aβ) plaques and intracellular neurofibrillary tangles. The production of Aβ, a decisive event in AD, is the result of the cleavage of amyloid precursor protein (APP), whose levels are high in AD.

APP has important developmental functions in cell differentiation and in the organization of synapses [54]. According to the Aβ hypothesis, AD begins with the abnormal processing of APP. Proteolysis of extracellular domains by sequential β-secretases and γ-secretases results in a family of peptides that form the β-amyloids (Aβ). Among these Aβ peptides, the more insoluble (Aβ_42_) has a propensity for self-aggregation into fibrils that form the senile plaques characteristic of AD pathology. Neurofibrillary tangles are composed of the tau-protein and in healthy neurons are integral components of microtubules, while in AD tau-protein becomes hyperphosphorylated and this phenomenon leads to the tangles binding to each other and forming tangled threads [55].

Brain inflammation is a pathological hallmark of AD, and we know that inflammation is a response to eliminate both the initial cause of cell injury as well as the necrotic cells and tissues resulting from the original insult. If tissue health is not restored, inflammation becomes a chronic condition that continuously erodes the surrounding tissues [55]. Inflammation clearly occurs in pathologically susceptible regions in brain AD, with increased expression of acute-phase proteins and pro-inflammatory cytokines [6,7,49,56-58]. The cells responsible for the inflammatory reaction are microglia, astrocytes, and neurons. These activated cells produce high levels of inflammatory mediators such as pro-inflammatory cytokines and chemokines, prostaglandins, leukotrienes, thromboxanes, coagulation factors, free radicals as reactive oxygen species and nitric oxide, complement factors, proteases and protease inhibitors, and C-reactive protein [49,58]. The hypothesis is that Aβ plaques and tangles stimulate a chronic inflammatory reaction [59]. Inflammatory mediators, in turn, enhance APP production and the amyloidogenic processing of APP to induce Aβ_42_ peptide production. These circumstances also inhibit the generation of a soluble APP fraction that has a neuroprotective effect [60,61]. On the contrary, Aβ induces the expression of pro-inflammatory cytokines in glial cells in a vicious cycle [62,63].

To date, the timing with which neuroinflammation is believed to influence AD is unknown. In particular, clinical and experimental evidence from different transgenic models has suggested that a pro-inflammatory process might precede plaque deposition [64]. A recent paper correlates the increased levels of C-reactive protein with the formation of senile plaques [65]. C-reactive protein has been shown to exist in two forms: the monomeric form, which has pro-inflammatory properties [66,67]; and the circulating pentamer form [68]. Authors have recently shown that the aggregated forms of Aβ plaques lead to the formation of the pro-inflammatory monomeric form of C-reactive protein, which exacerbates local inflammation [65].

There is currently much evidence suggesting the involvement of a systemic immune response in AD. Indeed, numerous investigations suggest that in addition to the central nervous system (CNS) cells, blood-derived cells can also be blamed for the inflammatory response and seem to accumulate in the AD brain [69-71]. Other studies have shown changes in the distribution and reactivity of immune cells in the blood [63,72-75]. Britschgi and Wyss-Coray have shown that there is communication between CNS and cells and factors involved in the systemic immune response [74]. In particular, neuroinflammation induces the efflux of proteins, such as Aβ, or inflammatory mediators from CNS across the blood–brain-barrier (BBB); this may cause systemic immune reaction and recruitment of myeloid or lymphocytic cells into the CNS.

Indeed, it is known that BBB has a “monitoring role” between the immune system and AD to protect the brain from the entry of macromolecules, like immunoglobulins, and cells, including immunocompetent cells. A recent assumption supposes that microvascular diseases, often associated with AD, microtraumas and inflammation could cause the abnormal permeability of the BBB. The consequence of this impairment is the anomalous presence of serum proteins in the cerebrospinal fluid and in the brain, including Aβ. In the brain Aβ can bind astrocytes, starting a degenerative and inflammatory process. Finally, autoantibodies bound to neurons can induce Aβ_42_ internalization and deposition, increasing brain damage [74,76].

Under physiological conditions T lymphocytes are few in the brain, although they are able to cross the BBB. The T-lymphocyte number increases in AD patients, especially in the hippocampus and temporal cortex. Herein, activated microglia increase the expression of MHC I and II, which allows the migration of T cells [76].

Communication between the CNS and the immune system in AD could thus influence both the lymphocyte distribution in the blood and the production of immune mediators [74]. Therefore, despite T cells being able to enter the brain tissue, it is also possible that T cells exert their effects without entering the CNS. Indeed, peripheral blood mononuclear cells (PBMCs) from AD patients produce higher levels of pro-inflammatory cytokines, such as IL-1β and IL-6, compared with PBMCs from control subjects [6,7,77]. Other studies have shown that Aβ stimulates macrophage inflammatory protein (MIP)-1α overexpression by peripheral T cells and its receptor CCR5 expression on brain endothelial cells necessary for T cells crossing the BBB [78]. Moreover, other altered immune parameters were documented, such as decreased percentages of naive T cells and an increase of memory T cells, an increased number of CD4^+^ T lymphocytes that lack the co-stimulatory molecule CD28, and a reduction of CD4^+^CD25^high^ regulatory T cells [79].

**Figure 1:**
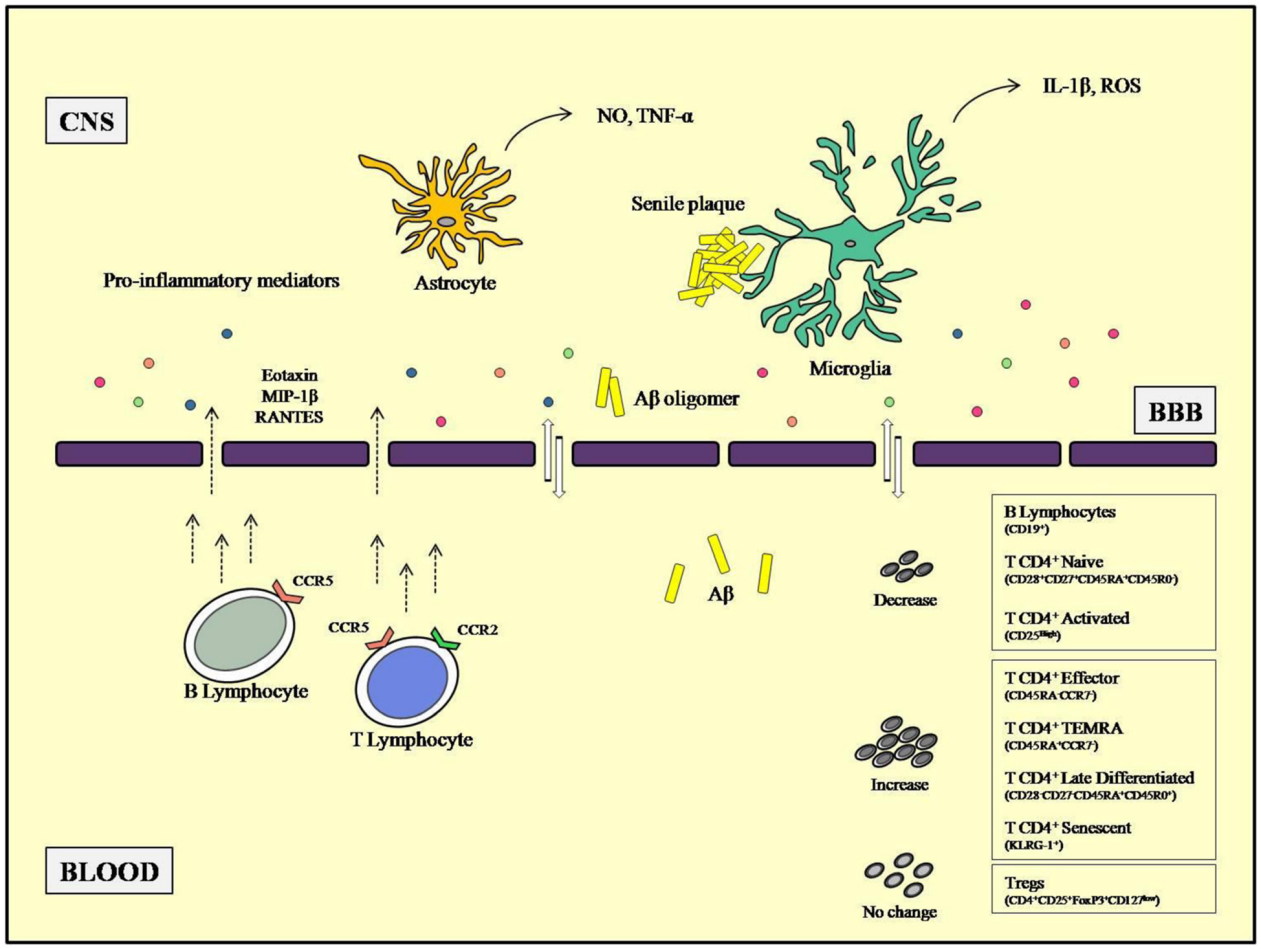
**Communication between the central nervous system and systemic immune responses in Alzheimer’s disease patients.** Inflammation clearly occurs in pathologically susceptible regions of the Alzheimer’s disease (AD) brain. Neurodegeneration and neuroinflammation can result in changes of central nervous system (CNS) proteins (for example, amyloid-beta (Aβ) peptide) or inflammatory mediators (acute-phase proteins and pro-inflammatory cytokines and chemokines) across the blood–brain-barrier (BBB). These CNS-derived proteins and mediators may induce systemic immune reactions and/or recruit lymphocytic cells into the CNS. The cells responsible for the inflammatory reaction in CNS are activated microglia and astrocytes. The hypothesis is that Aβ plaques and tangles stimulate a chronic inflammatory reaction. Other than CNS resident cells, blood-derived cells can also be blamed for inflammatory response and seem to accumulate in the AD brain due to the expression of chemokine receptors. The changes in lymphocyte distribution in the AD patient’s blood are also depicted.

Figure 1 shows the hypothesis that supports the involvement of the immune system in the pathogenesis of AD.

### Systemic immune profile in Alzheimer’s disease

At present a correct diagnosis of AD, characterized by pathological changes in the AD brain (that include neurological loss, extracellular amyloid plaques and intracellular neurofibrillar tangles), can be only evaluated by post-mortem autopsy, although a recent study [61] emphasized the role of soluble Aβ oligomers as a key factor responsible for the early pre-plaque formation. Activation of microglia occurs in the early stages of the disease, even before plaque formation, and is correlated with early cognitive deficits. As a consequence of the microglial activation and the deregulation of nerve grow factor metabolism, these authors have indicated matrix metalloproteinase-9 as a possible biomarker for signaling the early stages of ongoing CNS inflammation [61]. Another study has put in evidence the use of imaging techniques for early detection of glial activation prior to plaque deposition [80].

The evaluation of some modified parameters obtainable from the blood of patients could therefore be a goal for the research on AD.

The knowledge of the aforementioned systemic inflammation in AD patients has suggested a new research area that focuses on leukocyte modifications, as it would be desirable to have methods available that allow the use of peripheral blood from patients to identify “prognostic” or disease markers.

In this scenario, many authors have identified changes in lymphocyte distribution and in cytokine levels in the plasma of AD patients [75,79,81] that support the involvement of the immune system in AD. Many studies have reported alterations of both the innate and acquired immune system [74], although there are many discordant results (Table 2). Indeed, our group and others [63,82,83] have reported a decrease both in the percentage and the absolute number of total B cells from AD patients when compared with age-matched healthy controls. We did not observe any changes for the other main lymphocyte subpopulations [63]. On the contrary, Xue and colleagues have shown a significant reduction of CD3^+^ T cells, but no changes in CD4^+^ and CD8^+^ T-cell subsets [83]. Richartz-Salzburger and colleagues confirm the decrease of CD3^+^ and CD8^+^ T cells, but showed a slight increase of CD4^+^ cells [81]. Larbi and colleagues emphasized the dramatic changes within the CD4^+^ T-cell compartment, with a reduction of naïve CD4^+^CD45RA^+^CCR7^+^ and a simultaneous increase of effector memory CD4^+^CD45RA^-^CCR7^-^ T cells and of terminal effector memory RA CD4^+^CD45RA^+^CCR7^–^ T cells [79]. Again, the authors have demonstrated a reduction of CD4^+^CD25^high^ cells, potentially considered regulatory T cells [79].

**Table 2:** Main modifications of lymphocytes subpopulations between Alzheimer’s disease patients and age-matched controls

**Phenotype**	**Lymphocyte subpopulation**	**Changesin Alzheimer disease**	**Reference**
CD19^+^	Total B cells	Decrease	[82]
(percentage)			[83]
			[63]
CD19^+^	Total B cells	Decrease	[82]
(absolute number)			[63]
CD3^+^	Total T cells	No change	[63]
(percentage)		Decrease	[81]
			[83]
			
CD3^+^CD8^+^(percentage)	Cytotoxic T lymphocytes	No change	[63]
			[83]
		Decrease	[81]
CD3^+^CD4^+^	T-helper cells	No change	[63]
(percentage)			[83]
		Increase	[81]
CD3^+^CD4^+^CD45RA^+^CCR7^+^	Naïve CD4^+^ T	Decrease	[79]
(percentage)	cells		
CD3^+^CD4^+^CD28^+^CD27^+^CD45RA^+^CD45RO^-^	Naïve CD4^+^ T	Decrease	[75]
(percentage)	cells		
CD3^+^CD4^+^CD45RA^-^CCR7^-^	Effector memory	Increase	[79]
(percentage)	CD4^+^ T cells		
CD3^+^CD4^+^CD45RA^+^CCR7^-^(percentage)	Terminal effector memory RA cells	Increase	[79]
			
CD3^+^CD4^+^CD28^-^CD27^-^CD45RA^+^CD45RO^+^	Late differentiated	Increase	[75]
(percentage)	CD4^+^ T cells		
CD3^+^CD4^+^CD25^high^	Activated CD4^+^ T	Decrease	[79]
(percentage)	cells		
CD3^+^CD4^+^CD25^+^FoxP3^+^CD127^low^(percentage)	Regulatory T cells	No change	[75]
CD3^+^CD4^+^KLRG-1^+^(percentage)	Senescent CD4^+^ T cells	Increase	[75]

More recently, the use of larger numbers of surface markers confirmed the significant reduction of naïve CD4^+^ T cells, identified as CD4^+^CD28^+^CD27^+^CD45RA^+^CD45RO^-^ in AD patients, compared with age-matched controls and a contemporary increase of CD4^+^CD28^–^CD27^-^CD45RA^+^CD45RO^+^ late differentiated memory T cells [75]. The further evaluation of CD57 and KLRG-1, commonly considered senescence markers on these cells, has demonstrated a significant increase of late differentiated KLRG-1^+^CD4^+^ T cells in AD patients compared with age-matched healthy controls. No differences have been reported concerning CD57 expression on CD4^+^ T cells when comparing AD patients and their controls [75]. Moreover, the deep characterization of regulatory T cells as CD4^+^CD25^+^FoxP3^+^CD127^low^ has demonstrated no differences between the two groups studied, thereby revealing that the previously reported data [79] are referred to activated T cells (CD4^+^CD25^+^) instead of regulatory cells. Table 2 describes the reported data.

Regarding CD8^+^ T cells, no modifications are reported in AD patients when compared with their age-matched controls. Indeed, this might be due to the well-known role of CD8^+^ T cells in age-related changes strictly correlated with chronic cytomegalovirus infection, which is a feature common to almost all older people (as well as AD patients) [35-37].

### Aβ_42_ and *in vitro* peripheral blood mononuclear cell activation

A recent hypothesis suggests that persistent stimulation of the immune system by Aβ peptides leads to B-cell and T-cell responses, as well as to the release of inflammatory mediators.

Although the Aβ aggregates are mainly found in the brain amyloid plaques, the soluble forms, monomers and oligomers, predominate in the plasma where they may interact with the cells of the immune system [84].

Activation markers and chemokine receptors are overexpressed in unstimulated AD cells when compared with controls. This is evidence for the pro-inflammatory status of AD [6,7,85,86]. In this scenario, we have reported an *in vitro* response of T cells to recombinant Aβ_42_ (rAβ_42_). Indeed the CD69 activation marker is overexpressed in rAβ_42_-stimulated AD cells when compared with their controls [63]. Moreover, we have also reported an increased expression of the chemokine receptors CCR2 and CCR5 only on T cells of AD patients after *in vitro* stimulation by rAβ_42_, whereas B cells overexpress CCR5 after the same *in vitro* treatment. The modulated expression of these receptors might enhance the migration of lymphocytes across the brain microvascular endothelial cells [87,88]. Strictly related to the expression of chemokine receptors is the observation that peripheral T lymphocytes of AD patients produce higher MIP-1α levels than age-matched controls [78]. This observation, together with the expression of the MIP-1α receptor CCR5 on the human brain microvascular endothelial cells, might explain the migration of T cells and B cells across the BBB. Microglial cells also produce MIP-1α. It has been demonstrated that MCP-1 via CCR2, expressed on brain endothelial cells, contributes to increased brain endothelial permeability [74,78]. In contrast to these data, we did not observe any significant overproduction of MIP-1α in PBMCs *in vitro* stimulated by rAβ_42_. This discrepancy might be due to the different experimental systems used since the production/binding of MIP-1α *in vivo* or *in vitro* was assessed using human brain microvascular endothelial cells [78]. Moreover, in AD patients we and others [63,89] have demonstrated an increased production of RANTES, which is one of CCR5′s ligands (Table 3).

**Table 3:** **Cytokines, growth factors, chemokines and chemokine receptors on Alzheimer’s disease patients after *in vitro *** **stimulation**

	**Stimulated vs. unstimulated AD patients**	**Reference**
Cytokines		
IL-1β,IL-6,TNF-α,IL-1ra	Increase	[63]
IFN-γ	Increase	[63]
		[82]
IL-10	Decrease	[77]
	Increase	[63]
Growth factors		
GM-CSF,G-CSF	Increase	[63]
Chemokines		
Eotaxin,MIP-1β	Increase	[63]
RANTES	Increase	[89]
MIP-1α	No change	[63]
Chemokine receptors		
CCR2 and CCR5 on T cells	Increase	[63]
CR5on B cells	Increase	[63]

AD, Alzheimer’s disease; G-CSF, granulocyte colony-stimulating factor; GM-CSF, granulocyte–macrophage colony-stimulating factor; IL-1ra, IL-1 receptor antagonist; MIP, macrophage inflammatory protein.

The role of Aβ_42_ in the generation of an “inflammatory milieu” is also suggested by the observation that *in vitro* stimulation of PBMCs by rAβ_42_ induces the production of different chemokines and cytokines, rendering these cells active players in the inflammatory response in AD patients [63]. In fact, after an *in vitro* stimulation of PBMCs, AD patients have shown a significantly high production of the inflammatory cytokines IL-1β, IL-6, TNF-α and IFN-γ. We have also reported an increase of the anti-inflammatory cytokines IL-10 and IL-1 receptor antagonist, so we hypothesized that this situation might balance the overproduction of the above-described pro-inflammatory cytokines. As previously stated, however, there is an efflux of amyloid from CNS that can prime lymphocytes. Some authors have demonstrated a reduction of both pro-inflammatory and anti-inflammatory cytokines, hence assuming a general impairment of immune functions in AD patients, whereas others have demonstrated a decrease of IL-10, an increase of MIP1-α and an increase of IFN-γ, respectively [74,78,82,88]. Methodological differences (mitogen or Aβ stimulation) among the different studies, including inclusion criteria for both AD patients and healthy controls, might explain the great variability of data (Table 3).

Since monocytes are the main source of IL-6 and TNF-α and they possibly efficiently bind Aβ_42_ via CD36, the pattern of cytokine production observed by us is the one to be expected. Besides, we have previously demonstrated an increased expression of the scavenger receptor CD36 on monocytes from AD subjects in unstimulated and stimulated cultures that could be related to their efficient role to bind plasmatic Aβ which in turn causes the production of cytokines, chemokines, and reactive oxygen species, hence activating the signaling cascade necessary for cellular migration, adhesion, and phagocytosis [63].

In addition, the engagement of monocytes might render these cells more efficient in T-cell activation [90]. Some studies have suggested receptors for advanced glycosylation end products as possible candidates for the role of soluble Aβ receptors. These receptors have been found on CD4^+^ T-cell surfaces and are known to bind various molecules including Aβ; ligation of receptors for advanced glycosylation end products results in cell activation and inflammatory response [91]. Another possible receptor might be Toll-like receptor-4 [92,93], expressed on CD4^+^ T cells, for which the potentially modulatory effect upon ligation by Aβ may even be direct.

## Conclusions

Many modifications of immune and inflammatory systems have been reported in patients affected by AD. These changes might be the consequence of the overproduction of Aβ that can activate the blood cells, rendering them active producers of inflammatory mediators. On the contrary, the role of the genetic background namely the polymorphisms of genes involved in the immune-inflammation must be considered to fully elucidate the complex mechanisms that play a role in the generation of AD. Moreover, as a high proportion of women are affected by AD, especially at a very advanced age, it is important to consider the role played both by hormones and levels of education regarding the different propensity of males and females to develop disease. Fascinatingly, other important risk factors that could be related to the typical pro-inflammatory status of older people are environmental events in early life as well as childhood IQ.

## Abbreviations

Aβ: amyloid-beta; AD: Alzheimer’s disease; APP: amyloid precursor protein; BBB: blood–brain-barrier; CCR: chemokine receptor type; CNS: central nervous system; IFN: interferon; IL: interleukin; IQ: intelligence quotient; IRP: immunological risk phenotype; KLRG-1: killer cell lectin-like receptor subfamily G member 1; MHC: major histocompatibility complex; MIP: macrophage inflammatory protein; PBMC: peripheral blood mononuclear cell; rAβ_42_: recombinant amyloid-beta 42; RANTES: regulated upon activation, normal T-cell expressed, and secreted; TNF: tumor necrosis factor.

## Competing interests

The authors declare that they have no competing interests.

## Authors’ contributions

AM, MB, SB and GC-R wrote the first draft. Subsequent drafts were written by AM, who had the overall supervision of the review processing. All authors edited the paper and approved its final version.
